# Genomic insights into antibiotic resistance genes in *Leuconostoc citreum* strains isolated from artisanal buffalo milk curd in Bangladesh through whole-genome sequencing

**DOI:** 10.1128/mra.01289-23

**Published:** 2024-02-15

**Authors:** Mst. Umme Habiba, M. Nazmul Hoque, Shabbir Ahmed, Md. Tofazzal Islam, Gautam Kumar Deb, Md. Morshedur Rahman

**Affiliations:** 1 Department of Dairy and Poultry Science, Bangabandhu Sheikh Mujibur Rahman Agricultural University (BSMRAU), Gazipur, Bangladesh; 2 Molecular Biology and Bioinformatics Laboratory, Department of Gynecology, Obstetrics and Reproductive Health, Bangabandhu Sheikh Mujibur Rahman Agricultural University (BSMRAU), Gazipur, Bangladesh; 3 Institute of Biotechnology and Genetic Engineering, Bangabandhu Sheikh Mujibur Rahman Agricultural University (BSMRAU), Gazipur, Bangladesh; 4 Biotechnology Division, Bangladesh Livestock Research Institute, Savar, Bangladesh; 5 Institute of Food Safety and Processing, Bangabandhu Sheikh Mujibur Rahman Agricultural University (BSMRAU), Gazipur, Bangladesh; Queens College Department of Biology, Queens, New York, USA

**Keywords:** whole genome, multidrug resistance, *Leuconostoc citreum*, buffalo milk curd

## Abstract

We sequenced the genome of *Leuconostoc citreum* strains BSMRAU-M1L6 and BSMRAU-M1L13 isolated from artisanal buffalo milk curd in Bangladesh. The draft genomes of BSMRAU-M1L6 and BSMRAU-M1L13 are 1,869,891 and 1,890,611 bp, respectively, with 50.0× coverage (both) and 65 and 75 contigs, respectively.

## ANNOUNCEMENT

The European Food Safety Authority and the World Health Organization have issued recommendations to exclude bacterial strains carrying mobile genetic elements with antibiotic-resistance genes from use in feeds, food fermentations, and probiotics (1). *Leuconostoc* species are generally used to produce aroma during milk fermentation (2). Research has shown the benefits of *Leuconostoc* strains (3), with *Leuconostoc citreum* considered the most significant species (4).

Thirty-five pure colonies of *Leuconostoc* species were isolated from naturally fermented buffalo milk curd available in Bhola (22.19° N, 90.76° E) and Noakhali (22.87° N, 91.27° E) districts of Bangladesh. The culture-dependent isolation was conducted using modified plate count agar media containing glucose as a carbon source, supplemented with vancomycin, and incubated at 22°C for 5 days according to a previously published method (5). After being grown overnight in MRS broth at 22°C for 48 h, the genomic DNA was extracted from each pure bacterial colony using QIAamp DNA Mini Kit (QIAGEN, Hilden, Germany) (6) and then amplified by PCR using the universal primers 27F (5′‐AGAGTTTGATCCTGGCTCAG‐3′) and 1492R (5′-GGTTACCTTGTTACGACTT-3′). After performing partial 16S rRNA gene sequencing (7), 27 isolates were identified as *Leuconostoc citreum*. This study aimed to sequence the genomes of two *Leuconostoc citreum* strains, BSMRAU-M1L6 and BSMRAU-M1L13, and they were found to be multidrug-resistant (MDR; resistant to eight antibiotics) in disk diffusion tests according to CLSI 2021 (8). The Nextera DNA Flex Library Prep Kit (Illumina, USA) was used to generate libraries from 1 ng of DNA, extracted from BSMRAU-M1L6 and BSMRAU-M1L13 strains as described earlier. The whole-genome sequencing was performed using the Illumina MiSeq sequencer (Illumina, USA) with a 2 × 250 bp protocol (6, 9, 10).

Generated raw reads (BSMRAU-M1L6 = 9,552,456 bp, BSMRAU-M1L13 = 6,589,096 bp) were trimmed using Trimmomatic v0.39 to remove Illumina adapters, known Illumina artifacts, and phiX reads (11) and quality checked using FastQC v0.11.9 (12). Reads with Phred scores > 20 were assembled using SPAdes v.3.15.5 (13), and assembled genomes were quality checked through QUAST v.5.0.2 (14). Prokaryotic Genome Annotation Pipeline v6.4 (15) was used for genome annotation. We utilized ResFinder 4.0 (16), PlasmidFinder (17), PHASTER (https://phaster.ca/), and RAST FIGfams v.70 (18) databases to predict antimicrobial resistance genes, plasmid, prophages, and metabolic functions, respectively, in the draft genomes. Default parameters were used for all software unless otherwise stated.

Both BSMRAU-M1L6 and BSMRAU-M1L13 were MDR isolates, showing resistance against cephalexin, ciprofloxacin, gentamicin, nalidixic acid, nitrofurantoin, tetracycline, penicillin, and vancomycin. The draft assembly sizes of BSMRAU-M1L6 and BSMRAU-M1L13 were 1,869,891 bp and 1,890,611 bp, respectively. Further sequencing and assembly statistics of both genomes are given in Table 1. Both genomes had 206 metabolic features under different subsystem categories. The BSMRAU-M1L6 genome contained 1,803 protein-coding sequences (CDS) and 51 total RNA genes, while 1,811 CDS and 38 total RNA genes were predicted in the BSMRAU-M1L13 genome. Only two ARGs were predicted in both genomes conferring resistance to vancomycin (e.g., *van*T and *van*Y). The BSMRAU-M1L6 genome contained three CRISPR arrays and five prophages, whereas two CRISPR arrays and four prophages were identified in the BSMRAU-M1L13 genome (with 95% identity and 60% coverage). These data will furnish valuable insights for researching the functions of these strains.

**TABLE 1:** Genomic features of the *Leuconostoc citreum* BSMRAU-M1L6 and BSMRAU-M1L13 strains

Isolate	Genome coverage (×)	Genome size (bp)	No. of contigs	*N*50 value (bp)	GC content (%)	Genome accession no.	SRA accession no.
BSMRAU-M1L6	50.0	1,869,891	65	66,784	39.0	JAVUPW000000000	SRR25321897
BSMRAU-M1L13	50.0	1,890,611	75	66,662	38.9	JAVUPX000000000	SRR25321896

## Data Availability

The whole genome shotgun project of the *Leuconostoc citreum* strains BSMRAU-M1L6 and BSMRAU-M1L13 has been deposited in GenBank and the NCBI Sequence Read Archive (SRA) under BioProject accession number PRJNA982305. Individual accession numbers are listed in Table 1. The versions described in this paper are version JAVUPW000000000.1 and JAVUPX000000000.1.
